# Strychnia as an Antidote to Chloral

**Published:** 1870-08

**Authors:** 


### Strychnia as an Antidote to Chloral.

M. Liebreich, after a number of experiments upon rabbits, arrives at the conclusion that strychnia, administered hypodermically, acts as an antidote to poisonous doses of chloral.—*The Pharmacist.*

